# 

**Published:** 1855-10

**Authors:** 


### NEW BOOKS.

We have received since the issue of our last number the following works, which will be noticed at greater length in a subsequent number: Carpenter's Human Physiology; Bedford's Obstetrical Clinique; Dickson's Elements of Medicine; Smith on Leucorrhea.

